# Age-Specific Responses to Immersive Virtual Reality During Pediatric Venipuncture: Evidence from Routine Clinical Practice

**DOI:** 10.3390/healthcare14020173

**Published:** 2026-01-09

**Authors:** Domonkos Tinka, Mohammad Milad Shafaie, Péter Prukner, Márta Kovács

**Affiliations:** 1 Doctoral School of Economic and Regional Sciences, Széchenyi István University, 9026 Győr, Hungary; tinka.domonkos@sze.hu; 2 Multidisciplinary Engineering Doctoral School, Széchenyi István University, 9026 Győr, Hungary; 3 Digital Development Center, Széchenyi István University, 9026 Győr, Hungary; peter.prukner@ddc.sze.hu; 4 Faculty of Health and Sport Sciences, Széchenyi István University, 9024 Győr, Hungary; kovacsm1011@gmail.com; 5 Department of Paediatrics, Petz Aladár University Teaching Hospital of Győr-Moson-Sopron County, 9024 Győr, Hungary

**Keywords:** virtual reality, pediatric pain, venipuncture, developmental differences, gender differences, procedural distraction, clinical engagement

## Abstract

**Background/Objectives**: Virtual reality (VR) is increasingly used to reduce pain during pediatric needle procedures, but its effectiveness may vary by developmental stage and gender. This study evaluated whether immersive VR reduces venipuncture pain in children and adolescents and examined parent–patient agreement and gender-specific response patterns. **Methods**: A prospective nonrandomized clinical study was conducted within a hospital-based pediatric venipuncture service using an alternating 1:1 allocation sequence. Participants aged 4–18 years underwent venipuncture with either VR (*n* = 49) or standard care (*n* = 29). Procedural pain was measured using the Faces Pain Scale–Revised (FPS-R) with independent parent ratings. Analysis of covariance (ANCOVA) compared post-procedural FPS-R scores while adjusting for baseline pain. Exploratory age and gender-specific analyses were also performed. **Results**: VR led to a clear reduction in pain for children, even after adjusting for baseline scores (3.55 vs. 4.73; *p* = 0.003). Adolescents, however, reported similarly low pain in both groups (2.81 vs. 2.79; *p* = 0.60), and several mentioned that the PEGI 3 content felt too young for them, which likely limited how engaged they were. Among children, girls showed the most noticeable drop in pain, which matches the subgroup’s adjusted significance (*p* = 0.011). Parent–patient agreement was stronger in children (r ≈ 0.7–0.8) than in adolescents (r ≈ 0.4–0.5), and VR did not change this pattern. Most participants said they would choose VR again for future procedures. **Conclusions**: Immersive VR helped reduce venipuncture pain in children but had little effect in adolescents, underscoring the need for age-appropriate or more interactive VR content for older patients. Overall, these findings support using VR selectively as a distraction tool that fits the developmental needs of pediatric groups.

## 1. Introduction

Venipuncture is one of the most common invasive procedures in pediatric care, and both children and adolescents usually describe it as painful and upsetting. When this pain is not managed well, it can strengthen needle-related fear, make children less cooperative and even lead them to avoid healthcare during future visits [1]. Immersive virtual reality (VR) reduces procedural pain primarily through attentional distraction [2,3]. Unlike passive methods such as television, VR creates a computer-generated 360-degree environment that strengthens the sense of presence and draws attention away from the needle [4,5]. By simultaneously engaging visual and auditory channels, immersive VR increases cognitive load and limits the attentional resources available for processing discomfort, thereby reducing perceived pain and anxiety during needle procedures [6,7].

Current pediatric pain guidelines recommend developmentally appropriate non-pharmacologic strategies such as preparation, coaching and distraction to reduce distress during needle procedures [8]. Immersive VR aligns with these recommendations, and clinical trials, as well as systematic reviews, report reductions in pain or anxiety during needle-related procedures across pediatric populations [7,9,10]. Some outpatient studies also indicate that VR reduces anticipatory anxiety before venipuncture, which may contribute to its increasing use in routine care [11]. Despite these promising findings, effect sizes vary notably. Many studies recruit wide pediatric age ranges without analyzing the Child and Adolescent groups separately, even though attention control, coping skills and responsiveness to distraction differ between these developmental stages. This is the first limitation in the existing literature. Most VR venipuncture studies report overall pain outcomes and do not assess whether age influences the effectiveness of immersive distraction [12]. Some authors propose that children may engage more readily with cartoon-based content, whereas adolescents often require greater interactivity or cognitively demanding environments for distraction. There is also evidence that gender may influence emotional engagement with immersive media [3].

A second gap in the literature relates to the inconsistent use of dual pain reporting. Parent–patient agreement provides meaningful insight into how families interpret procedural discomfort and how accurately caregivers estimate a child’s pain. Evidence outside the VR field indicates that parents typically estimate pain more accurately in the Child group than in the Adolescent group, who may mask distress or rely on more advanced regulatory strategies [13]. Few studies utilizing VR research in the field have explored parent–patient agreement, or whether this agreement differs by age or gender; therefore, there is a notable methodological gap in the literature. Several prior trials have demonstrated that immersive VR is an effective method of reducing procedural pain when compared to traditional distraction strategies, with many studies reporting stronger effects for pediatric participants, with emerging support for gender differences in effectiveness [5,9,10]. Building on this theoretical work, exploring whether age and gender impact the analgesic VR response during venipuncture is important. Despite evidence supporting the effectiveness of VR as a distraction method, nearly all studies to date have not provided subgroup-specific responses, which leaves clinicians uncertain which pediatric subgroup of patients would benefit most from immersive VR.

The present study addresses these gaps by evaluating a pragmatic immersive VR approach during routine venipuncture in a clinical pediatric setting. Pain outcomes were compared between VR and standard care while examining differences between the Child and Adolescent groups. Gender related patterns and parent–patient agreement were also assessed to clarify whether developmental or demographic factors influence the response to VR. This study provides new age-specific and gender-specific evidence on how immersive VR performs in real-world clinical practice and helps identify which pediatric subgroups benefit most from distraction-based interventions during venipuncture.

In summary, this study compared VR and standard care during pediatric venipuncture and examined age and gender related patterns. Immersive VR significantly reduced pain in the Child group, whereas Adolescents showed minimal change. These findings clarify which pediatric subgroups benefit most from immersive distraction during routine clinical procedures.

## 2. Materials and Methods

### 2.1. Study Design and Setting

**Figure 1:**
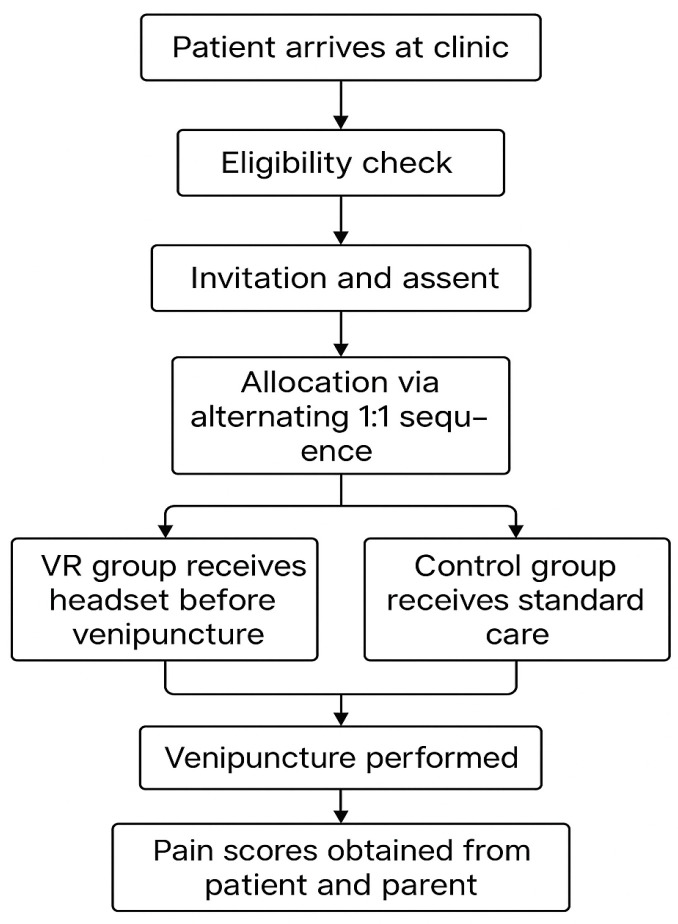
Procedural workflow and allocation sequence for the clinical venipuncture study.

This prospective clinical study was conducted in a high-volume pediatric clinic where venipuncture is routinely performed for diagnostic purposes. Children and adolescents scheduled for clinically indicated blood sampling were consecutively approached during regular clinic hours. Participants were allocated to the VR or control groups using an alternating one-to-one sequence that integrated with the clinical workflow and allowed uninterrupted patient turnover. No sedative or analgesic medication was administered solely for research purposes. Apart from introducing the VR headset in the intervention group, all elements of routine care, including preparation, positioning and procedural steps, followed standard clinic practice to preserve ecological validity. The procedural workflow and allocation method are summarized in Figure 1.

The study was conducted within a hospital-based pediatric venipuncture service as part of routine clinical care. Information regarding inpatient versus outpatient status was not recorded as a separate variable and therefore could not be examined analytically.

### 2.2. Participants and Age Classification

Eligible participants were children and adolescents aged 4 to 18 years who were able to understand and complete the FPS-R. Recruitment occurred on a rolling basis, and caregivers provided informed consent with child assent when appropriate. In addition to written informed consent obtained from parents or legal guardians, informed assent was obtained from all age-appropriate children and adolescents. Assent was provided after a brief, age-adapted explanation of the venipuncture procedure and the VR intervention, emphasizing voluntariness and the right to withdraw at any time without consequences. The inclusion of informed assent reflects current ethical standards in pediatric research and supports participant autonomy during clinical procedures. Exclusion criteria included significant cognitive impairment that prevented comprehension of the pain scale, major uncorrected visual or auditory deficits that interfered with VR use and urgent medical conditions requiring rapid venipuncture without the possibility of distraction. Patients with previous adverse reactions to VR, motion sickness or sensory hypersensitivity were also excluded at clinician discretion. Participants were categorized into two age groups based on developmental criteria commonly used in pediatric pain research: Child group (4–11 years) and Adolescent group (12–18 years) [14]. This classification is commonly used to approximate differences in cognitive development, attention regulation, coping strategies and responsiveness to immersive distraction. Demographic variables, including age, gender and clinical indication for venipuncture, were recorded for each participant.

Age-based grouping was determined a priori to reflect broad developmental stages commonly referenced in pediatric pain research. However, this categorization should be considered a conceptual and exploratory framework rather than an empirically validated construct within the present dataset.

### 2.3. VR Intervention

**Figure 2:**
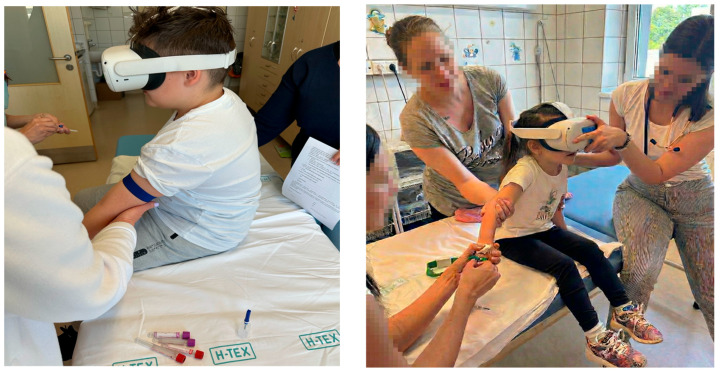
Clinical photographs of the VR intervention during pediatric venipuncture. Both images were captured by the research team during routine blood sampling with caregiver permission, illustrating the immersive headset setup used in the study in both inpatient and outpatient clinical contexts.

The VR condition used the Meta Quest 2, a standalone headset with integrated processing that does not require a smartphone or external computer. All VR participants viewed the same 360-degree animated short film, *Henry*, classified PEGI 3, which depicts a cartoon hedgehog’s birthday celebration. The content is fully immersive yet noninteractive, providing consistent passive distraction for all participants and reducing variability in user interaction. The runtime of approximately eight minutes was adequate for nearly all venipuncture procedures. The VR intervention consisted of a single, passive, child-oriented animated content that was shown to all participants regardless of age. While this approach supported standardization in a clinical setting, it may have limited engagement among adolescents and constrained the interpretability of null findings observed in this group. No timing data were recorded for either group, and the duration of headset exposure did not influence clinical decision-making. An illustrative example of the VR intervention during venipuncture is shown in Figure 2.

### 2.4. Measurement Instruments and Data Collection

The primary outcome was self-reported procedural pain measured immediately after venipuncture using the FPS-R, scored from 0 to 10. Parents independently rated their child’s pain on a 0-to-10 visual analogue scale (VAS), providing an additional perspective on perceived procedural discomfort. The post-procedure questionnaire also included items related to anxiety, perceived helpfulness of VR and willingness to use VR in future procedures. Additional descriptive data included the number of needle insertion attempts, staff-rated procedural difficulty and the participant’s preferred method for future blood draws. Demographic information and clinical indication for venipuncture were extracted from the medical record. No physiological measures were collected, and no sedation or analgesics were used unless clinically indicated. No topical anesthetics or sedative agents were used in either group. The number of puncture attempts was recorded descriptively but not included as a covariate, as it reflects procedural outcome rather than a pre-procedural confounder. Staff-rated difficulty was not analyzed due to the absence of a standardized scale. Prior needle fear/phobia and time on task were not formally assessed.

The rationale for using the FPS-R for baseline assessment, rather than a dedicated anxiety scale, was to ensure consistency in the measurement tool and to simplify the task for pediatric participants within a routine clinical workflow. In clinical settings, children often conceptualize their anticipatory distress in terms of expected pain, making a visual pain scale a pragmatic surrogate for pre-procedural anxiety when formal anxiety instruments are not feasible.

### 2.5. Statistical Analysis

Descriptive statistics summarized demographic and clinical variables. The primary analysis compared postprocedural FPS-R scores between the VR and control groups using ANCOVA. The previous FPS-R score was obtained retrospectively by asking patients and their parents to recall the pain intensity of the most recent prior venipuncture experience. This assessment was completed after the current procedure and was used as a covariate to adjust for baseline differences in prior pain experience. Postprocedural FPS-R was the dependent variable; group was the fixed factor and previous FPS-R was included as a covariate to adjust for baseline differences. The ANCOVA model followed the standard formulation:

$$Y_{ij}=\mu +\tau_{i}+\beta (X_{ij}-\overset{-}{X})+\varepsilon_{ij}$$

where $Y_{ij}$ denotes the adjusted postprocedural FPS-R for participant j in group i, $\tau_{i}$ represents the group effect, $X_{ij}$ is the previous FPS-R score, β is the regression coefficient and $\varepsilon_{ij}$ is the residual error term. This formulation aligns with the established ANCOVA methodology described in [15,16].

Separate ANCOVA models were estimated for the Child and Adolescent groups to account for developmental differences in distraction responsiveness. All ANCOVA models were estimated using complete-case analysis; participants with missing values in the dependent variable (postprocedural FPS-R) or the covariate (previous FPS-R) were excluded from the corresponding model. Exploratory gender-based analyses used the same model structure but were not powered for confirmatory inference. For ANCOVA models, effect sizes were reported as partial eta-squared (η^2^p). For the primary age-group ANCOVA, we additionally report 95% confidence intervals for the adjusted mean difference. The assumption of homogeneity of regression slopes was evaluated prior to the interpretation of adjusted effects.

Pain-change scores were calculated as:

ΔPain=Previous FPS-R−Current FPS-R

consistent with standard pre–post procedural pain estimation methods described in [17]. All statistical tests were two-tailed with a significance threshold of *p* < 0.05. Analyses were performed using standard statistical software. No corrections for multiple comparisons were applied because subgroup analyses were exploratory. Statistical analyses were conducted using Python (version 3.11) with the SciPy and NumPy libraries.

## 3. Results

### 3.1. Sample Characteristics

Seventy-eight participants were included in the final analysis, with forty-nine allocated to VR and twenty-nine to standard care. The Child group comprised 37 participants and the Adolescent group comprised 41. Gender distribution was similar across conditions, with 45 girls and 33 boys overall. Mean age, gender composition and clinical indications for venipuncture were comparable between the VR and control groups. Previous venipuncture experience did not differ systematically between conditions. Baseline previous pain was higher in the Child VR group than in the Child control group, which supported the use of ANCOVA in all primary analyses. Full baseline characteristics are shown in Table 1.

### 3.2. Patient-Reported Pain by Age Group

**Figure 3:**
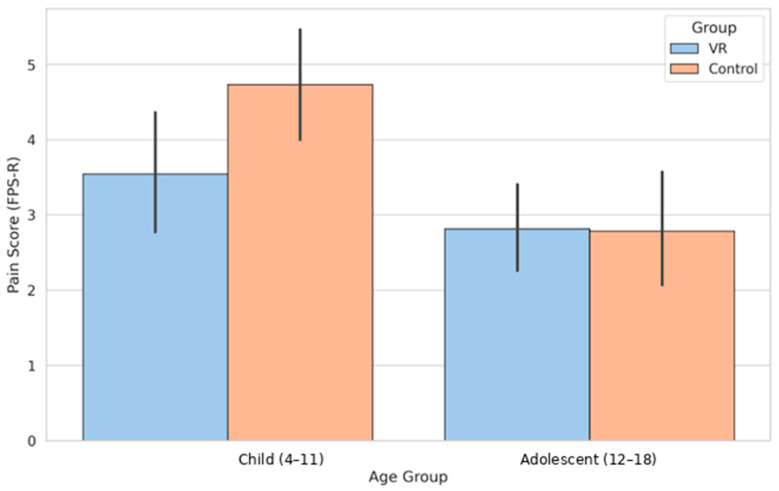
FPS-R scores for Children and Adolescents in VR and Control conditions.

Procedural pain differed by age group, and the response to VR was not uniform. Children who used VR reported lower pain compared with those receiving standard care, a pattern visible in the raw scores and illustrated in Figure 3.

- Child group:
- Mean FPS-R scores were 3.55 for VR and 4.73 for standard care. Because previous FPS-R scores were higher in the Child VR subgroup, ANCOVA was applied to adjust for baseline differences. In the adjusted model, group remained a significant predictor of current pain (F (1, 28) = 10.58, *p* = 0.003). This ANCOVA was conducted on complete cases (*n* = 31), demonstrating that the VR effect persisted after controlling for baseline pain. After adjustment for Previous FPS-R, children in the VR group reported lower postprocedural pain scores than those receiving standard care (adjusted mean difference = −1.18, 95% CI −1.92 to −0.44, η^2^p = 0.27, *p* = 0.003). This subgroup analysis was exploratory in nature and was not adjusted for multiple comparisons; therefore, the findings should be interpreted with appropriate caution.
- Adolescent group:
- Mean FPS-R scores were 2.81 for VR and 2.79 for standard care. The ANCOVA model confirmed the absence of a group effect (F (1, 38) = 0.28, *p* = 0.60). This ANCOVA was conducted on complete cases (*n* = 41). This suggests that passive, noninteractive VR content may not provide sufficient cognitive engagement to alter pain perception in adolescents. Low baseline pain in this group may also limit measurable change. This analysis was exploratory and not adjusted for multiple comparisons, and the absence of a group effect should be interpreted cautiously in light of limited statistical power.

### 3.3. Patient-Reported Pain by Gender

**Figure 4:**
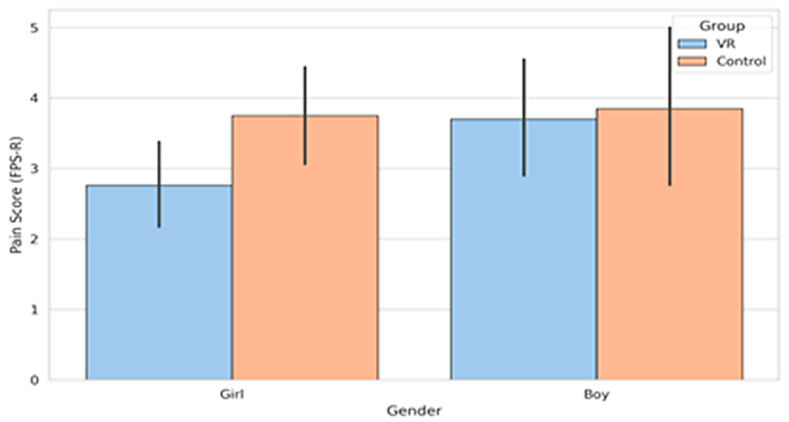
FPS-R scores by gender for VR and Control conditions.

Gender-based analyses indicated different patterns of VR responsiveness. Girls showed a clearer distinction between VR and control scores, whereas boys demonstrated smaller and more variable differences. These patterns are shown in Figure 4.

- Girls:
- Raw pain scores were lower in the VR group, and this difference persisted after adjusting for baseline pain. The ANCOVA model showed a significant group effect (F (1, 40) = 7.10, *p* = 0.011). This ANCOVA was conducted on complete cases (*n* = 43). These results indicate that the intervention produced a measurable reduction in procedural pain among girls.
- Boys:
- Raw mean FPS-R scores were similar between groups, and this similarity remained after adjustment. The ANCOVA model showed no significant group effect (F (1, 26) = 1.75, *p* = 0.20). This ANCOVA was conducted on complete cases (*n* = 29). These findings indicate that the analgesic effect of VR was not consistent across genders. Although this pattern aligns with previous observations of higher emotional engagement among girls, the subgroup analysis was exploratory and should be interpreted cautiously due to sample size. Gender-based subgroup analyses were exploratory and not adjusted for multiple comparisons and should be interpreted cautiously.

### 3.4. Agreement Between Parent and Patient Ratings

**Figure 5:**
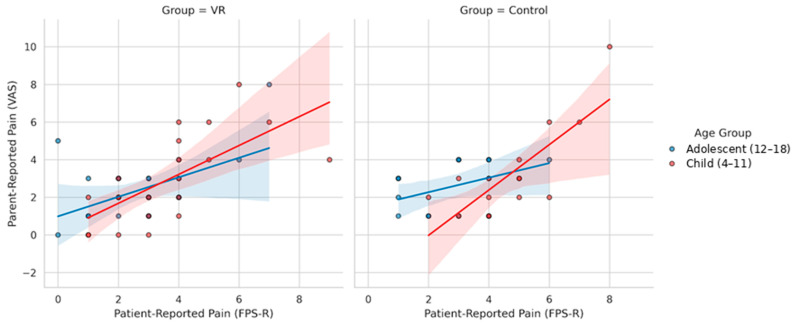
Scatterplots illustrating the relationship between patient-reported FPS-R scores and parent-reported pain ratings in the VR and control groups, stratified by age group (Child vs. Adolescent). Blue indicates Adolescents (12–18) and pink indicates Children (4–11); solid lines show the fitted regression lines with shaded 95% confidence intervals.

Parent–patient agreement was quantified using Pearson correlation coefficients. In the VR group, correlations were strong in children (r = 0.68, 95% CI 0.36–0.86, *p* < 0.001) and moderate in adolescents (r = 0.52, 95% CI 0.18–0.75, *p* = 0.005). In the control group, correlations were similarly strong among children (r = 0.78, 95% CI 0.45–0.92, *p* < 0.001), whereas agreement was weaker and not statistically significant in adolescents (r = 0.44, 95% CI −0.12–0.79, *p* = 0.12). Formal comparisons using Fisher’s r-to-z transformation showed no statistically significant differences in correlation strength between VR and control conditions or between age groups. These associations are illustrated in Figure 5.

In the Child group, correlations commonly ranged from r = 0.7 to 0.8, reflecting close agreement between caregiver observations and self-reported pain. This pattern is consistent with previous findings showing that caregivers tend to judge the distress of children more accurately, as this group generally shows clearer behavioral cues during venipuncture [10]. In adolescents, correlations were lower, typically around r = 0.4 and 0.5, likely because they rely more on internal coping strategies and show fewer observable signs of discomfort. The introduction of VR did not meaningfully change these age-related differences, as agreement remained stronger in the Child group under both conditions.

### 3.5. Change in Pain by Age Group and Gender

**Figure 6:**
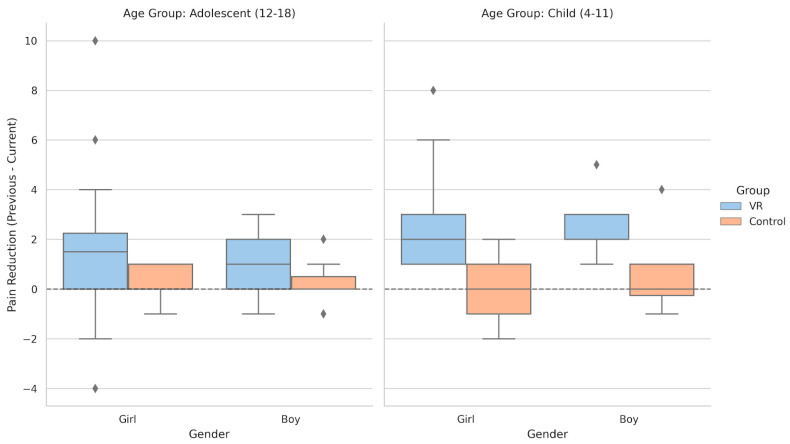
FPS-R pain-change scores by age and gender.

Pain-change scores offered additional insight into subgroup differences by indicating whether the intervention altered perceived discomfort beyond baseline levels. These scores represented the difference between previous and current FPS-R values and helped clarify patterns not captured by raw means. The distribution of pain-change scores across age and gender groups is presented in Figure 6.

Girls in the Child group demonstrated the most pronounced reductions, with several decreases of two or more FPS-R points, which is consistent with the ANCOVA results and suggests greater responsiveness to immersive distraction in this subgroup. Boys in the Child group showed smaller and more variable changes, with median values close to zero. Among adolescents, median change scores were near zero across genders and conditions, indicating that neither VR nor standard care produced measurable alterations in perceived pain at this developmental stage. Overall, these findings show that VR-related pain reduction was concentrated in a specific subgroup rather than uniformly distributed across the sample.

### 3.6. Preference for VR in Future Procedures

**Figure 7:**
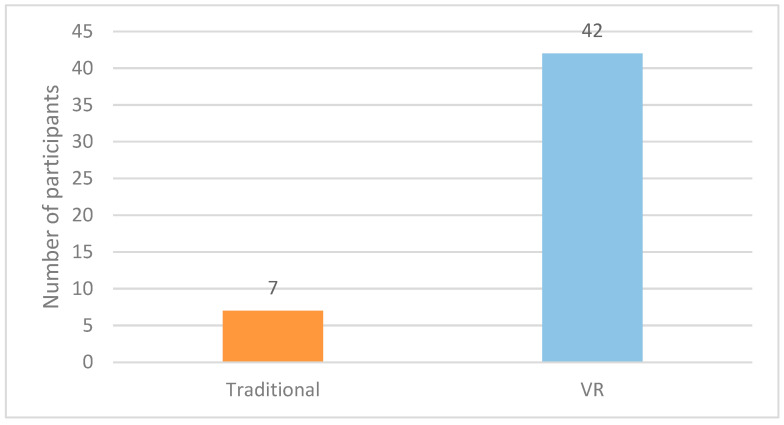
Participants’ preferred method for future venipuncture (VR vs. Traditional).

Participant preferences offered additional insight into the acceptability of the intervention during routine venipuncture. After the procedure, each participant indicated whether they would choose VR or the traditional method for future visits, and these preferences are summarized in Figure 7.

Forty-two participants (85.7%) said they would choose VR again for future procedures, while seven preferred the traditional approach. Interest in VR stayed high even among those who showed little or no change in their pain scores, which suggests that elements like immersion, comfort or simply the novelty of the experience may shape preference independently of the measured pain reduction. Overall, VR was easy to use in the clinical workflow, well tolerated and generally viewed as a positive option for supporting children during procedures.

In addition to pain outcomes, secondary questionnaire items related to acceptability were collected and are reported descriptively. Other items were intended to provide contextual insight rather than analytic efficacy outcomes.

### 3.7. Safety and Tolerability

VR-related safety and tolerability outcomes were documented during the procedure. Among participants who used the VR headset (*n* = 49), one participant (1/49, approximately 2%) reported mild vertigo. In addition, one participant discontinued VR immediately due to discomfort attributed to mild headset vibration, and two participants declined VR before the procedure due to a prior history of motion sickness/vertigo. No other adverse events, such as nausea or persistent dizziness, were reported.

### 3.8. Summary of Key Results

Together, these results indicate that the analgesic benefits of passive immersive VR were primarily observed in younger pediatric patients, whereas no significant differences were detected in the adolescent group. Gender-specific analyses suggested greater VR-related pain reduction among girls than boys, while parent–patient agreement was consistently stronger in children than adolescents across conditions. Pain-change scores supported these subgroup patterns, and acceptability of VR remained high regardless of pain reduction magnitude. The intervention was well tolerated, with no serious adverse events reported.

## 4. Discussion

This study found that immersive VR mainly helped children with venipuncture pain. Children who used VR reported lower FPS-R scores than those who received standard care (3.55 vs. 4.73), and this difference stayed significant even after adjusting for their baseline pain (*p* = 0.003). In contrast, adolescents showed almost identical pain levels in both groups (2.81 vs. 2.79), and the adjusted model confirmed that VR did not make a measurable difference for them (*p* = 0.60). These patterns match earlier work showing that children usually respond better to distraction techniques during needle procedures [1,9,10]. The greatest improvement was seen among girls in the Child group, suggesting that both age and gender may influence how well immersive distraction works [5,18].

This age-specific difference may be attributed to the nature of the VR content used in this study. While passive distraction is often sufficient for younger children, adolescents may benefit more from VR environments that provide higher levels of interaction and cognitive engagement. The child-oriented, passive content used in our protocol may have provided limited cognitive challenge for the adolescent group. Accordingly, the absence of a measurable effect in this subgroup may reflect a mismatch between content design and developmental stage rather than a definitive lack of responsiveness to VR-based distraction. These observations highlight the importance of age-tailored and interactive VR design when considering distraction strategies for pediatric populations.

This pattern is consistent with findings from earlier randomized trials. Previous studies have shown that VR can help lower pain and procedural distress during pediatric venipuncture [9,10], and similar reductions in needle-related discomfort have been noted in younger children across other clinical settings as well [19]. Recent work also shows benefits for procedural anxiety and overall procedural tolerance, using both immersive and educational VR formats [13,14], while additional studies report improvements when VR is used as part of procedural preparation [20]. Multicenter findings support consistent improvements in comfort across pediatric environments [21], and reports from other procedure types further illustrate VR’s broader value as a distraction-based method [22]. Collectively, these data emphasize that immersive distraction may be particularly well suited for younger pediatric patients.

The stronger response among girls in the Child group may reflect differences in attention control or emotional engagement with immersive content. Younger children tend to rely more on external distraction approaches, and narrative-based VR may facilitate a more complete attentional shift for some subgroups. Prior work has suggested that emotional engagement can contribute to VR-related analgesic effects [5]. Although the subgroup analyses in the present study were exploratory and not powered for confirmatory conclusions, and no adjustment for multiple comparisons was applied, the observed trends should be interpreted as hypothesis-generating rather than confirmatory and warrant replication in adequately powered studies.

Patterns of parent–patient agreement were consistent with established developmental findings, with stronger correlations in children (r ≈ 0.7 to 0.8) and more modest associations in adolescents (r ≈ 0.4 to 0.5) [13]. The use of VR did not reduce this concordance, suggesting that while immersive distraction may influence internal pain perception, it does not obscure observable behavioral cues that caregivers rely on during procedures. This stability is important in clinical care settings where caregiver judgments often guide supportive actions. Consistent with previous clinical observations, acceptance of VR was high among participants [23].

A methodological consideration in this study was the higher baseline previous-pain score in the Child VR subgroup. Adjustment through ANCOVA addressed this imbalance, and VR remained a significant predictor of reduced pain, indicating that the effect was not solely due to regression toward the mean. The alternating allocation sequence, while compatible with a high-throughput clinical workflow, introduces potential allocation or performance bias and should be taken into account when evaluating causal interpretation.

Although the present study relied on self-reported pain outcomes and caregiver ratings, the inclusion of objective engagement measures (e.g., physiological or behavioral indicators of immersion) may help clarify the mechanisms underlying age-specific responses to VR distraction, particularly among adolescents.

In conclusion, immersive VR provided the clearest analgesic benefits for the Child group, with the strongest response observed among girls, while adolescents showed minimal measurable change with passive VR content. These developmental patterns underscore the need for age-appropriate distraction strategies and support further investigation into interactive, tailored VR environments and objective engagement measures in larger randomized studies.

## 5. Limitation

There are limitations to this study that should be taken into account when interpreting the results. First, there was no standardized way to record when procedures were initiated; additionally, the length of time spent using VR was only about eight minutes, and will most likely not be the same duration as the control group’s procedures. Second, the patients were assigned to treatment or control conditions on an alternating 1:1 basis, which did not involve concealment. Participants were allocated using an alternating 1:1 sequence without allocation concealment or blinding, which may introduce allocation and performance bias and limit causal inference. Because allocation was predictable and neither staff nor participants were blinded, expectations regarding group assignment could have influenced behavior and pain reporting, further limiting causal interpretation of the observed effects.

Another limiting factor is the constraints related to measurement. Several adolescents mentioned that the content of PEGI 3 was too geared toward children, which may have reduced their engagement with the intervention and contributed to the minimal pain-related effect seen in this group. Although these perceptions were informal, participants’ evaluation of content suitability was not assessed systematically using validated measures. As a result, these observations should be interpreted as anecdotal rather than empirical findings. Future studies should incorporate standardized tools to assess perceived content appropriateness and engagement across developmental stages. Accordingly, the adolescent subgroup findings should be interpreted with caution and considered hypothesis-generating, as limited engagement with the passive, child-oriented content may have constrained the observed effects in this age group.

This study relied only on the self-report FPS-R and parental VAS scores, and because no physiological indicators such as EEG, HRV or eye-tracking were collected, it was not possible to match subjective pain ratings with any objective signals. Some potential confounders could not be fully addressed, including the lack of standardized difficulty ratings, formal assessment of prior needle fear and procedural timing data.

Although FPS-R is conventionally used to assess pain, the retrospective assessment of previous FPS-R may partially reflect anticipatory distress rather than pain per se and may be subject to recall bias. This should be considered when interpreting adjusted analyses, and future studies should incorporate validated measures of anticipatory anxiety. This limits how clearly we can understand what might be driving the pain-reducing effect of VR.

Data limitations also need to be noted. The sample was small, which made it hard to test interaction effects with enough statistical strength, and the subgroup estimates were not very stable because of that. Baseline pain scores were also higher in the Child VR group, which raises the possibility of regression toward the mean. We adjusted for this using ANCOVA and the VR effect still appeared, but some residual confounding is still possible. A larger sample with more balanced baseline values would help give clearer subgroup conclusions.

Generalizability is another key issue to consider. Because inpatient versus outpatient status was not systematically recorded, potential setting-related differences in pain perception or response to VR could not be examined. As procedural experiences may vary across clinical contexts, the generalizability of these findings beyond hospital-based care should be interpreted with caution. Future studies conducted across multiple care settings should explicitly record clinical context and evaluate its potential influence on VR effectiveness.

This study focused specifically on venipuncture in a pediatric clinical setting, so the findings may not extend to other procedures or to different pediatric populations. Although VR has shown analgesic benefits for adolescents in other situations, such as burn wound care [24], the version used here was passive and child-oriented and does not represent the full range of VR approaches available in clinical practice. Even with these limits, the real-world clinical environment and the inclusion of both child and parent pain ratings support the ecological validity of the findings and reflect how VR may function during routine venipuncture in everyday practice.

Building on these limitations, several directions for future research emerge. Studies employing age-tailored and interactive VR content may help clarify whether adolescent null effects reflect content mismatch rather than limited responsiveness to immersive distraction. Larger cohorts allowing continuous age modeling, alongside validated measures of engagement and acceptability, would further refine developmental interpretations. Finally, multicenter investigations spanning inpatient and outpatient settings could strengthen clinical generalizability and inform implementation of VR-based distraction as a scalable support tool in routine pediatric procedures.

## 6. Conclusions

This study showed that immersive VR reduced venipuncture pain primarily in the Child group, where participants using VR reported lower FPS-R scores than those receiving standard care, and this difference remained significant after adjustment for baseline previous pain. Adolescents showed little variation across conditions, and gender patterns indicated that the most pronounced reduction occurred among girls within the Child subgroup. Parent–patient agreement remained stable, and most participants indicated willingness to use VR in future procedures, suggesting that the intervention was acceptable and easy to integrate into routine clinical practice.

These findings should be interpreted alongside several methodological constraints, including the modest sample size, baseline imbalances and the use of an alternating allocation sequence. Even so, the results suggest that immersive distraction may be most effective for developmentally younger patients. Future research should evaluate age-appropriate or interactive VR environments for adolescents, include physiological markers of engagement and employ larger randomized designs to clarify which pediatric subgroups benefit most from VR during needle procedures.

## Figures and Tables

**Table 1:** Baseline characteristics of participants in the VR and control groups.

Variable	VR (*n* = 49)	Control (*n* = 29)
Age (years), mean ± SD	11.73 ± 3.73	12.14 ± 3.44
Girls, *n* (%)	29 (59.2%)	16 (55.2%)
Boys, *n* (%)	20 (40.8%)	13 (44.8%)
Child, *n* (%)	22 (44.9%)	15 (51.7%)
Adolescent, *n* (%)	27 (55.1%)	14 (48.3%)
Previous FPS-R, mean ± SD	5.02 ± 2.62	3.81 ± 1.92

## Data Availability

The data supporting the findings of this study are not publicly available due to patient privacy restrictions, but they are available from the corresponding author upon reasonable request.

## Abbreviations

The following abbreviations are used in this manuscript:

VR	Virtual reality
FPS-R	Faces Pain Scale–Revised
VAS	Visual analogue scale
